# Nitrogen a Necessity in the Development of the Teeth

**Published:** 1891-08

**Authors:** W. H. Whitslar

**Affiliations:** Youngstown, O.


### COMMUNICATIONS.

### Nitrogen a Necessity in the Development of the Teeth.

BY W. H. WHITSLAR, M.D., D.D.S., YOUNGSTOWN, O.

Destitute of taste, odor, or color, and a non-supporter of combustion, nitrogen one of the elements of created beings presses forward as one of the prime factors of our existence.

Pertaining to physiological life nitrogen supplies a vast territory in constructive and reconstructive measures; pertaining to its usage by man in medicine its benefits are by no means meagre.

Man is ushered into this world a delicate but wonderfully constructed being to be assisted by a tender and careful nurse, Dame Nature; but her efforts are often thwarted by human agency through lack of care or knowledge.

The infant, as soon as it is delivered from its mother's womb, becomes surrounded by a different medium than that in which it heretofore existed; it is now æriform. Oxygen is no longer supplied through the blood of the mother, but this æriform medium, commonly called atmospheric air, gives a larger quantity than has heretofore been given to the child. The quantity is limited, however, by nitrogen, and such is the provision of nature or else there would be a rapid consumption of tissues and a short life would be expended. One part of oxygen is limited by four parts of nitrogen being mixed with it but *not* in a state of combination. It is a remarkable fact that either upon the high mountain or upon the low plain this property of oxygen and hydrogen exists. Nitrogen is, however, in a state of combination as a necessary constituent of the tissues of the human body. It is found in various forms and in considerable quantity. In the bones of a child thirty-three per cent. is ossein, which is nitrogenized material. The ossein in the bones of woman is 27.61 per cent. and in man 20.42 per cent. according to Bibra.

In the blood there is 1 to 2 per cent. of nitrogen (Foster), and in lymph 1.12 to 1.82 per cent. 100 volumes of lymph (Hammarsten).

In the excreta of the body is found considerable nitrogen, some two or three hundred grains being discharged daily by the kidneys alone. This is accounted for because quite a portion of the food entering the body is not fully digested. From this great loss it is evident that the supply must come from some outside source (1) the atmosphere and (2) the food. Experiment has shown that it is not consumed or absorbed in the act of respiration, but a certain amount is swallowed with the food and absorbed by the gastro-intestinal mucous membrane.

Food containing nitrogen must then be practically the only source of the constant supply which is essential to maintain the body in a normal condition.

Nitrogenous foods are those contaning albumen chiefly, examples of which are white of eggs, casein, legumin, gluten and syntonin. Collagens are nitrogenous foods and are bodies that yield gelatin with ossein as an example. Ossein is a large per cent. of the organic matter of a tooth.

Vegetables yield albumen. Those rich in albumen and nitrogen are cabbage, asparagus, cress and mushrooms. While it is observed that nitrogenized food is favorable to the occurrence of inflammation, and scurvy seems to be caused by an absolute excess of nitrogen in the food, yet children frequently suffer from deficiency of nitrogen.

In anæmia and chlorosis nitrogen is needed and in such cases the teeth come late and do not resist decay. Addition of oatmeal, barley, or rice in the food often bring about a marked improvement. Good food is an essential part of the treatment in all cases where there is a deficiency of tissues, and especially so in children since they need more during the years of their development than at the adult age. Hence, due allowance of meat, oatmeal, corn, beans, peas, and other foods containing nitrogen should be given to construct healthy tissues of the human body. Among these tissues the teeth are of great importance and nitrogen is a necessary factor of their development.

